# Inebriety: Its Source, Prevention and Cure

**Published:** 1897-12

**Authors:** 


INEBRIETY: ITS SOURCE, PREVENTION AND CURE. By CHARLES FOLLEN PALMER. Sexto-decimo, pp. 109. Price, 50 cents. New York: Fleming H. Revell Company, 112 Fifth avenue.

This book is written from the moral standpoint rather than from the material. If the reader of this volume expects to find an easy way of reforming the drunkard, or valuable formulæ for relieving the alcoholic, he will be disappointed. The chapter on cure deals with the psychic side. Instead of drugs and hychotherapy, we find a discussion of the following precepts : (1) Be a man whatever you do. (2) A trained will-power is an essential to self-preservation. (3) An unselfish wife not always best for a weak husband. (4) The first step is to persuade an inebriate to enter an asylum for rest and security for a period not exceeding three months. (5) Purgation of evil thoughts of others as essential. (6) Evils of a life of ease. As will be seen from these propositions which are maintained in a philosophical spirit, the book is that of a moral idealist. As an example of this style of thought it is elevating, but not a practical help. J. W. P.

